# Psychological Resilience Enhances the Orbitofrontal Network in the Elderly With Mild Cognitive Impairment

**DOI:** 10.3389/fpsyt.2019.00615

**Published:** 2019-08-29

**Authors:** Sang Joon Son, Bumhee Park, Jin Wook Choi, Hyun Woong Roh, Na-Rae Kim, Jae Eun Sin, Haena Kim, Hyun Kook Lim, Chang Hyung Hong

**Affiliations:** ^1^Department of Psychiatry, Ajou University School of Medicine, Suwon, South Korea; ^2^Department of Biomedical Informatics, Ajou University School of Medicine, Suwon, South Korea; ^3^Office of Biostatistics, Ajou University School of Medicine, Suwon, South Korea; ^4^Department of Radiology, Ajou University School of Medicine, Suwon, South Korea; ^5^Department of Brain Science, Ajou University School of Medicine, Suwon, South Korea; ^6^Department of Psychiatry, The Catholic University of Korea College of Medicine, Yeouido St. Mary’s Hospital, Seoul, South Korea

**Keywords:** resilience, functional connectivity, fMRI, orbitofrontal cortex, elderly

## Abstract

**Background:** It has been suggested that maintaining the efficient organization of the brain’s functional connectivity (FC) supports neuroflexibility under neurogenerative stress. This study examined psychological resilience-related FC in 112 older adults with mild cognitive impairment (MCI).

**Methods:** Using a resting-state functional magnetic resonance imaging (fMRI) approach, we investigated reorganization of the orbitofrontal gyrus (OFG)/amygdala (AMG)/hippocampus (HP)/parahippocampal gyrus (PHG) FC according to the different levels of resilience scale.

**Results:** Compared with the low resilient group, the high resilient group had greater connectivity strengths between the left inferior OFG and right superior OFG (*P* < 0.05, Bonferroni corrected), between the right inferior OFG and left PHG (*P* < 0.05, Bonferroni corrected), and between the right middle OFG and left PHG (false discovery rate < 0.05).

**Conclusion:** Psychological resilience may be associated with enhancement of the orbitofrontal network in the elderly with MCI.

## Introduction

Pathology processes of major neurocognitive disorder begin before the onset of clinical symptoms. However, many patients remain free of symptoms for a considerable period despite a significant neurodegeneration and brain volume loss The concept of brain “resilience” has emerged to explain individuals’ ability to tolerate disease-related pathology in the brain without developing clinical symptoms or signs (1). Of the many aspects of brain resilience, interest in psychological resilience and its mechanism has increased in terms of major neurocognitive disorder prevention.

To address this issue, it is required to assess functional network in brain. Network resilience derives from the efficient arrangement of connections between brain regions (2, 3). It has been suggested that maintaining the efficient organization of the brain’s functional connectivity (FC) supports neuroflexibility under neurogenerative stress (3–5). For instance, brain regions’ FCs associated with negative emotional processing and regulation, and self-referential function could be modulated and affected by antidepressant treatment (6).

More specifically, it is worth noting the relations between the orbitofrontal gyrus (OFG)-related functional network and psychological resilience (2). OFG functional activity is known to play a mediating role in subjective well-being (7). It was also reported that resilient group had greater connectivity between the OFG and amygdala (AMG) (8). Feng et al. suggested that patients with depression showed weaker functional connectivity links between the medical OFG and the parahippocampal gyrus (PHG)/medial temporal lobe, which are involved in pleasant feelings and rewards with memory systems (9).

Considering this background, the present study was designed to examine psychological resilience-related FC in the elderly with mild cognitive impairment (MCI) accompanied by depression and anxiety symptoms. Using a resting-state functional magnetic resonance imaging (fMRI) approach, we investigated linear trends of the OFG/AMG/hippocampus (HP)/PHG FC according to the level of resilience scale. In addition, given the modulatory roles of psychological resilience, we examined whether these FCs were associated with depression, anxiety, and cognitive functions.

## Methods

### Participants

We recruited participants over the age of 60 with MCI accompanied by depression and anxiety symptoms from the geriatric community mental health center in Suwon, Republic of Korea. One hundred twelve subjects with a mean age of 73.78 ± 5.76 years (76.80% women) were recruited. All participants were diagnosed with depressive disorder by psychiatrists a year ago at the time of study enrollment and had taken antidepressants. Inclusion criteria were (a) MCI criteria proposed by Petersen et al. (10), (b) Clinical Dementia Rating (CDR) of 0.5 (11), (c) Clinical Global Impression-Severity (CGI-S) score below 4 points and not worse than 1 year ago, and (d) the use of antidepressants and anxiolytics at stable dosage for at least 6 weeks prior to study entry without any recommendation for changes in medication. Given the characteristics of older adults from geriatric community mental health center, participants might have chronic or residual affective symptoms, but they were clinically stable on affective symptoms. We excluded those who met the following criteria: (a) a history of severe psychiatric disorder (mental retardation, schizophrenia, bipolar disorder, and other dementia); (b) a history of neurological disorder, such as brain tumor, intracranial hemorrhage, subarachnoid hemorrhage, epilepsy, hydrocephalus, encephalitis, metabolic encephalopathy, or other neurologic conditions that could interfere with the study; (c) a history of significant hearing or visual impairment; and (d) a history of physical illnesses that could interfere with the study.

### Psychological Resilience Measurement

The Brief Resilience Scale (BRS) is a simple measurement consisting of six questions. Three questions are positive, and three are negative. Each score is given 1 to 5, and negative scores are added inversely. The higher the total score, the more psychological the resilient state. This scale was validated for Korean population (Cronbach’s alpha = 0.6, test–retest reliability = 0.62) (12, 13). In order to ensure sufficient number of neuroimaging analysis for each group, BRS was treated as a categorical variable based on tertiles. Subjects were divided into three groups based on BRS: from the lowest to 12 points was referred to as *2 group* (*n* = 62); from 13 to 23 points was *1 group* (*n* = 21); 24 points or more was *0 group* (*n* = 29).

### Measurement of Other Clinical Variables

Depressive symptoms were measured using the Montgomery–Asbergo Depression Rating Scale (MADRS) (14, 15). The MADRS consists of 10 items of depressive symptoms in seven stages from zero to six points. Beck Anxiety Inventory (BAI) was used to evaluate anxiety symptoms. The BAI is a self-rating tool to distinguish anxiety from depression. It has a total of 21 questions and is rated 0–3 for each question (16). Both scales indicate that depression or anxiety increases as scores increase. Cognitive functions were assessed using the Mini Mental State Examination (MMSE), Stroop Test-color reading, Seoul Verbal Learning Test (SVLT)-delayed recall, Digit Span-backward, and CDR (17).

### MRI Data Acquisition and Preprocessing

Resting-state fMRI (Rs-fMRI) was performed at the beginning and end of the study at Ajou University Hospital. All MRI acquisitions were performed with a 3.0-Tesla Philips scanner (Intera Achieva, Philips, Medical Systems, Best, The Netherland) located at Ajou University Hospital. For resting-state fMRI, gradient echo-planar imaging (EPI) sequence was collected (repetition time (TR) = 2,000 ms, echo time (TE) = 30 ms, flip angle = 90°, field of view (FOV) = 220 × 220 mm^2^, voxel size = 2.75 × 2.75 × 3 mm^3^, volumes = 176). Rs-fMRI data were acquired while participants lying down and resting with eyes closed, without focusing on any specific thoughts, and without sleeping. High-resolution T1-weighted images were acquired from each subject using a magnetization-prepared rapid acquisition gradient echo (MPRAGE) pulse sequence (TR = 2,000 ms, TE = 4 ms, flip angle = 8°, FOV = 220 × 220 mm^2^, voxel size = 1 mm^3^).

### Clinical Data Analyses

Descriptive statistics were used to explore the data. Categorical variables between resilience groups were compared using the chi-square test, while continuous variables were compared using an analysis of variance. SPSS software version 22.0 (SPSS Inc., Chicago, IL, USA) was used for all statistical analyses.

### fMRI Data and FC Analyses

Resting-state fMRI data preprocessing was conducted using statistical parametric mapping (SPM12, http://www.fil.ion.ucl.ac.uk/spm/, Wellcome Trust Centre for Neuroimaging, London, UK) (18). After the first five scans were discarded due to some stability issues, all EPI data were preprocessed by correcting for the delay in the acquisition time between different slices and correcting for head motion by realignment of all consecutive volumes to the first image of the session. The realigned images were co-registered to T1-weighted images, which were used to spatially normalize functional data into a template space using nonlinear transformation. We did not conduct spatial smoothing on the resting-state fMRI data to avoid inflation of local connectivity and clustering.

**Figure 1:**
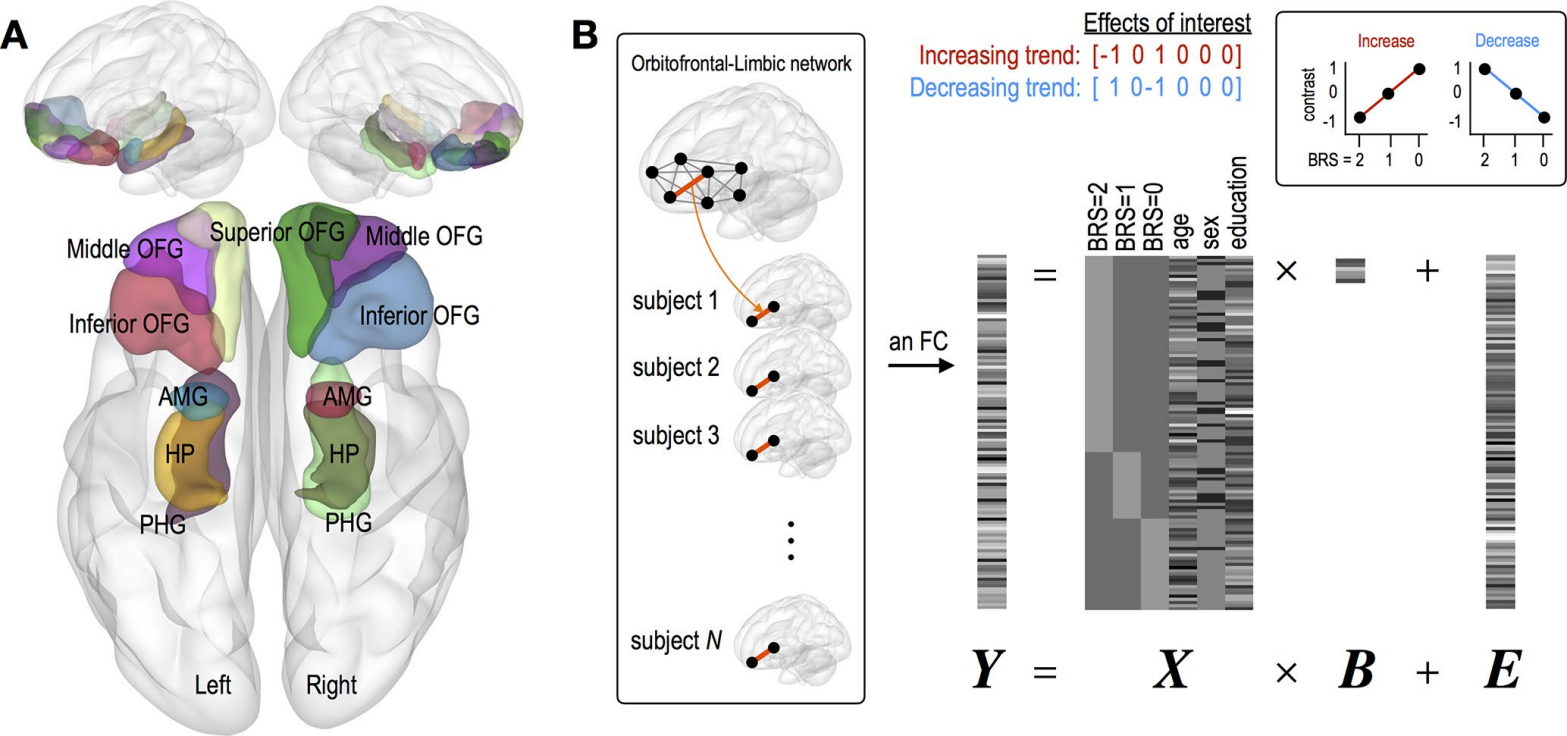
Description of brain regions of interest and a general linear modeling used in the current study. **(A)** Twelve regions of interest consist of the bilateral inferior/middle/superior OFG, HP, PHG, and AMG. **(B)** A general linear modeling where individual FC is tested with two contrast vectors representing linear increasing and decreasing trend separately. Variables of age, sex, and education were *z*-scored. Abbreviation: OFG, orbitofrontal gyrus; HP, hippocampus; PHG, parahippocampal gyrus; AMG, amygdala.

For the current study, we selected 12 regions of interest (ROIs) as the bilateral inferior/middle/superior OFG, HP, PHG, and AMG (**Figure 1A**), which were defined using automated anatomical labeling (AAL) atlas (19). Regional mean fMRI time series, extracted from the 12 regions, were temporally processed through (a) regressing out effects of six rigid motions and their derivatives, and three principal components of the white matter and the cerebrospinal fluid mask segmented using SPM12; (b) spike detection and despiking based on four times of the median absolute deviation; and (c) band-pass filtering (0.01–0.1 Hz) (20–23). Finally, we estimated inter-regional FCs among 12 regions using Pearson correlation coefficients, which were converted into *z*-scored maps with Fisher’s *r*-to-*z* transformation.

This study is aimed to investigate where individual FC shows linearly increasing or decreasing trend according to BRS scales. For this purpose, we used a general linear model, in which we designed the first three regressors (BRS 2, 1, and 0 groups) indicating different groups and the next three regressors (age, sex, and education year) including nuisance covariates (**Figure 1B**). With this design matrix, the linear trend for each FC was examined using two contrast vectors, that is, C = [−1 0 1 0 0 0] for check increasing FC and C = [1 0 −1 0 0 0] for check decreasing FC. In order to exclude the effect of changes in brain connectivity caused by depressive and anxiety symptoms, the same analyses were conducted in groups with scores of MADRS (≥34, *N* = 52; 20 to 33, *N* = 48; ≤19, *N* = 12) (24) sand BAI (≥32, *N* = 20; 27 to 32, *N* = 11; 22 to 26, *N* = 15; ≤21, *N* = 66) (25).

To detect FCs showing significant trend, we applied three threshold levels of false discovery rate (FDR) < 0.05, FDR < 0.2, and *P* < 0.05 (Bonferroni corrected) for multiple-comparison correction with the number of connections. Note that FDR control levels in the range of 0.1∼0.2 are originally known to be acceptable for multiple-comparison correction (26).

For FCs exhibiting significant linear trends, we further investigated whether such trends of connection strengths are related to MADRS, MMSE, and BAI scores or not. This study was conducted using a newly defined design matrix, where these clinical variables were separated for each group (BRS 2, 1, and 0 groups) and three nuisance covariates were included as well (**Figure 3A**). All analyses for resting-state fMRI were performed using MATLAB-based custom software.

## Results

### Clinical Characteristics of Participants

Demographic information and clinical data are summarized in **Table 1**. There were statistical differences in MADRS and BAI scores according to resilience groups.

**Table 1:** Demographic and clinical information.

Variable	Resilience level			χ^2^ or *F*	*P*-value
	High (BRS 0) (*n* = 29)	Moderate (BRS 1) (*n* = 21)	Low (BRS 2) (*n* = 62)		
**Age (years)**	74.79 ± 5.43	74.81 ± 5.31	72.95 ± 6.01	1.44	0.243
**Sex (female)**	19 (65.50)	15 (71.40)	52 (83.90)	4.15	0.126
**Education (years)**	7.14 ± 4.32	5.88 ± 4.78	5.70 ± 3.93	1.19	0.307
**BRS**	24.00 ± 0.01	18.48 ± 2.84	11.52 ± 1.65	538.95	< 0.001
**MADRS**	27.38 ± 10.38	31.90 ± 6.55	32.47 ± 8.43	3.52	0.033
**BAI**	14.14 ± 9.03	19.05 ± 10.92	21.94 ± 10.54	5.73	0.004
**MMSE**	24.03 ± 3.49	22.57 ± 2.60	23.37 ± 3.79	1.06	0.352
**CGI-S**	3.40 ± 0.72	3.76 ± 0.54	3.70 ± 0.61	2.85	0.062
**SVLT-delayed recall (z score)**	−1.06 ± 1.44	−0.43 ± 1.02	−0.92 ± 1.14	1.75	0.179
**Stroop Test—color reading (z score)**	−0.49 ± 1.36	−1.44 ± 1.50	−0.79 ± 1.44	2.49	0.088
**Digit Span-backward (z score)**	−0.54 ± 1.09	−1.14 ± 1.25	−0.59 ± .97	2.47	0.089

Values are mean ± standard deviation or n (%). BRS, Brief Resilience Scale; MADRS, Montgomery–Asberg Depression Rating Scale; BAI, Beck Anxiety Inventory; MMSE, Mini Mental State Examination; CGI-S, Clinical Global Impression-Severity; SVLT, Seoul Verbal Learning Test.

### Resilience and OFG/AMG/HP/PHG FC

**Figure 2:**
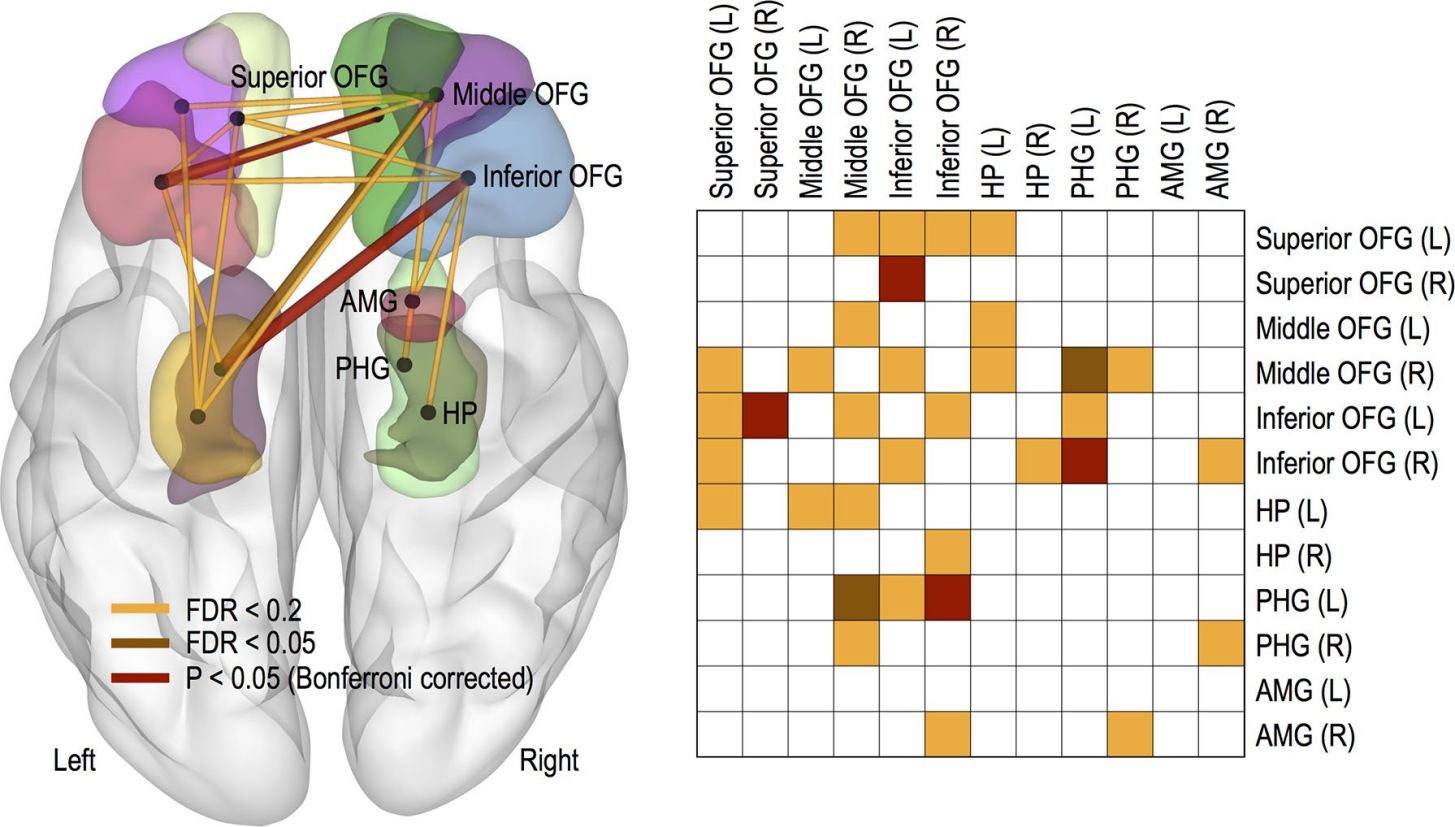
Functional connections showing significant increasing trends. Thicker links represent more significant connections and color-coded differently for three threshold levels of FDR < 0.05, FDR < 0.2, and *P* < 0.05 (Bonferroni corrected). Abbreviation: OFG, orbitofrontal gyrus; HP, hippocampus; PHG, parahippocampal gyrus; AMG, amygdala; L, left; R, right.

Compared with the low resilient group (BRS 2 group), the high resilient group (BRS 0 group) had greater FC strength, as follows: the left inferior OFG and right superior OFG (*P* < 0.05, Bonferroni corrected), the right inferior OFG and left PHG (*P* < 0.05, Bonferroni corrected), and the right middle OFG and left PHG (FDR < 0.05). However, we did not find any reduced FC strength in the high resilient group compared with the low resilient group. See **Figure 2** for more details and a complete list of our results.

Meanwhile, no changes in OFC FC were found according to the group of MADRS score. It was also observed that only FC between the left middle OFG and left HG increased in group with low BAI score (FDR < 0.2).

### Different Associations Between Cognitive/Depression/Anxiety Symptoms and OFG/AMG/HP/PHG FC According to Resilience Group

**Figure 3:**
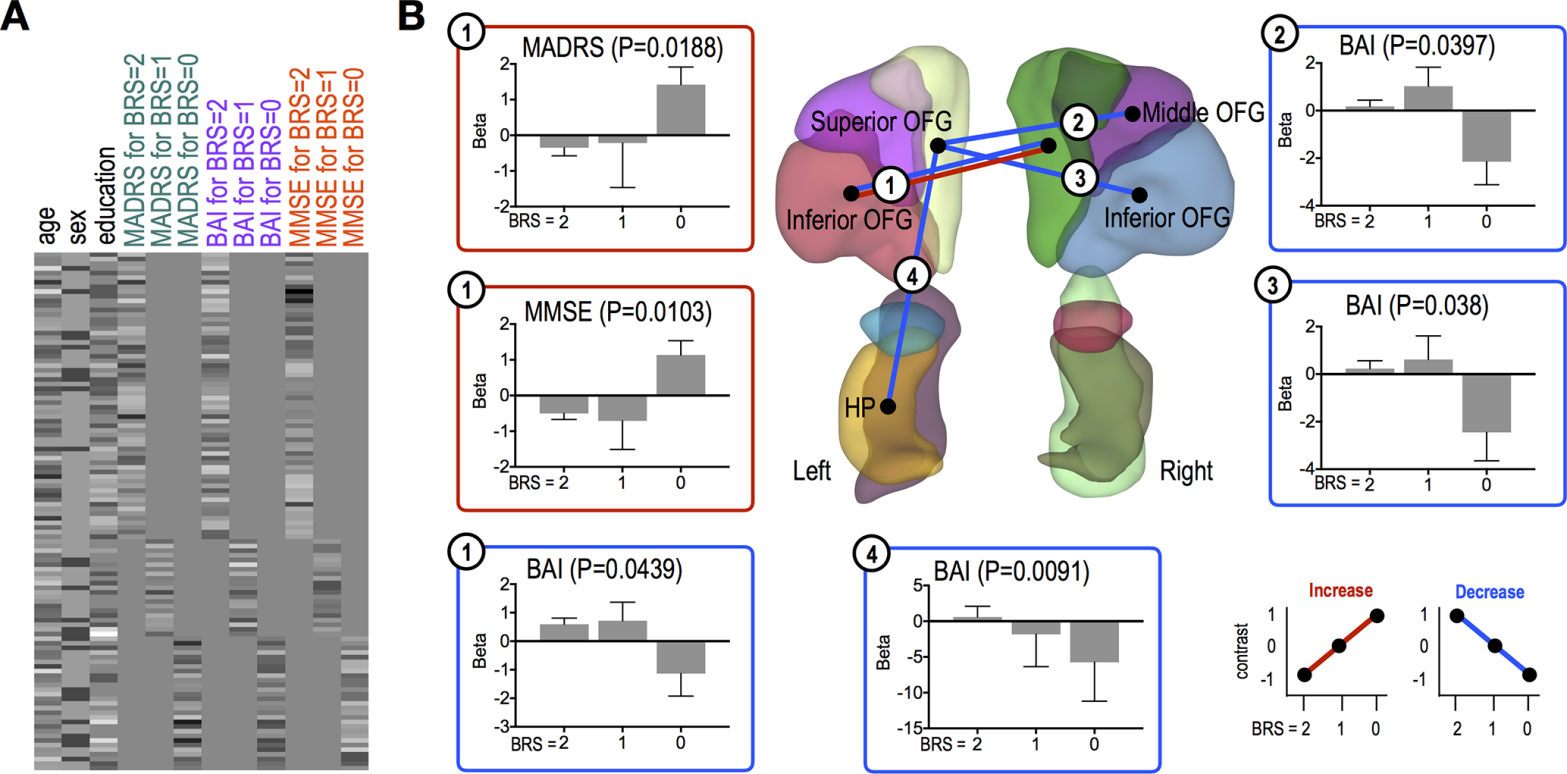
Functional connections showing significant association trends with clinical scores. **(A)** A design matrix used in association study with clinical scores. All variables were *z*-scored before separating to each group (BRS = 2, 1, and 0). **(B)** Functional connections showing significant trends for associations between the connections and clinical scores. In each bar plot, beta values on the vertical axis represent regression coefficients, and error bars imply standard error of the betas. Red and blue lines/boxes represent FCs and variables exhibiting significantly increasing and decreasing trends, respectively. Abbreviation: OFG, orbitofrontal gyrus; HP, hippocampus; PHG, parahippocampal gyrus; AMG, amygdala.

MMSE and MADRS scores were significantly positively correlated with FC strength between the left inferior OFG and right superior OFG (MMSE, *P* = 0.0103; MADRS, *P* = 0.0188) in the high resilient group (**Figure 3B**). However, we found significant negative correlations between BAI score and connectivity strength between the left inferior OFG and right superior OFG (*P* = 0.0439), the left superior OFG and right middle OFG (*P* = 0.0397), the left inferior OFG and right superior OFG (*P* = 0.038), and the left superior OFG and left HP (*P* = 0.0091) in the high resilient group (i.e., BRS 0 group) (**Figure 3B**).

**Figure 4:**
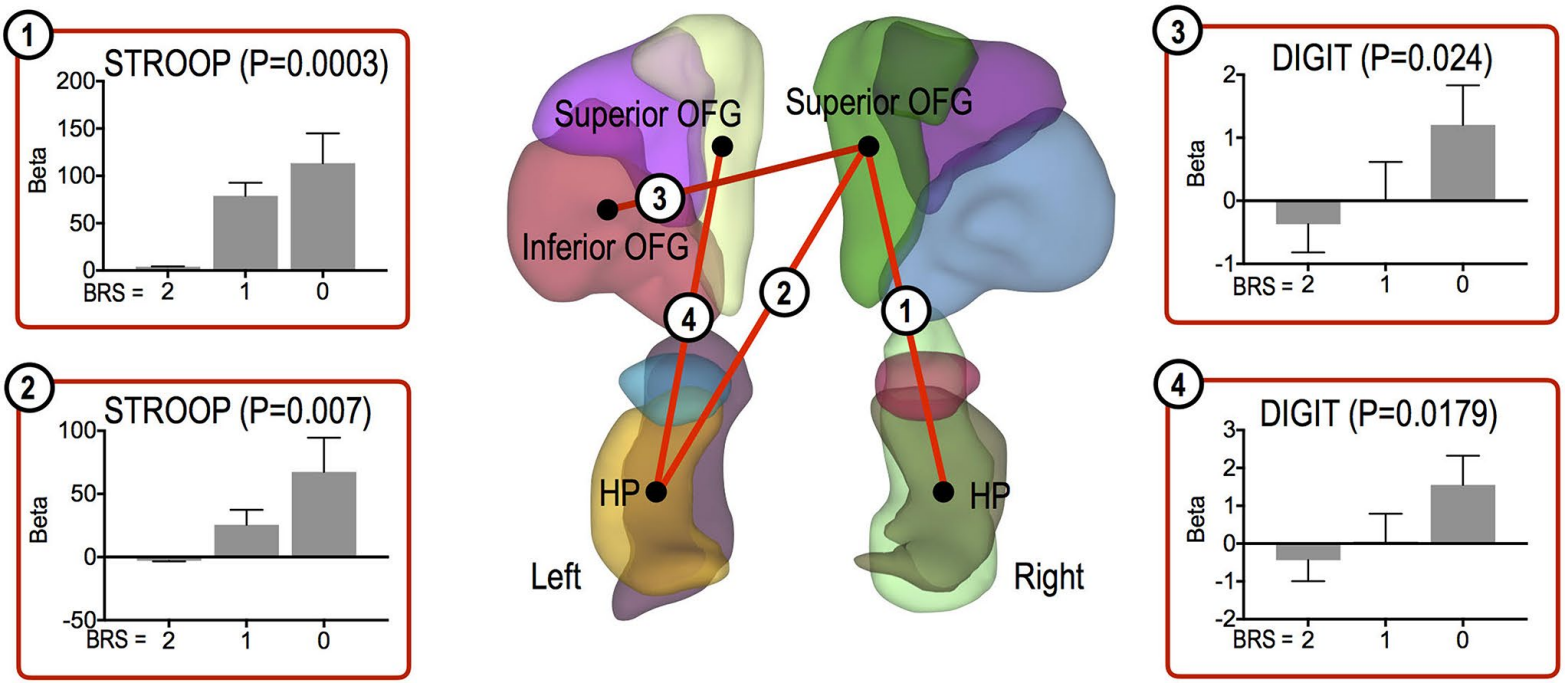
Functional connections showing significant association trends with cognitive function test scores. Functional connections showing significantly increasing trends for associations between the connections and cognitive scores. In each bar plot, beta values on the vertical axis represent regression coefficients, and error bars imply standard error of the betas. Abbreviation: OFG, orbitofrontal gyrus; HP, hippocampus.

Other cognitive function tests were significantly positively correlated with FC strength in the high resilient group, as follows: the right superior OFG and left HP (Stroop Test, *P* = 0.007), the right superior OFG and right HP (Stroop Test, *P* = 0.0003), the left superior OFG and left HP (Digit Span, *P* = 0.0179), and the right superior OFG and left inferior OFG (Digit Span, *P* = 0.024) (**Figure 4**).

## Discussion

The main finding is that psychological resilience may be associated with increased orbitofrontal network in the elderly with MCI. Brain circuits with greater FC strength in the high resilient group involved the OFG and PHG, which are implicated in the reward-related memory system (9). We also observed enhanced interconnectivity between OFG subregions as the resilience level increased.

Recent literature indicated that activation of the brain’s reward system could mitigate subsequent stress responses in humans, suggesting reward pathways as a mechanism for promoting psychological resilience (27). It was proposed that the connections between the medial OFG and HP/PHG provided a route for reward/emotion-related information (28). Therefore, based on our findings, it could be assumed that these FCs are involved in rewards system through positive emotional memory, which could be associated with psychological resilience under stress.

Meanwhile, enhanced interconnectivities between OFG subregions in proportion to the degree of resilience were found in this study. Previous neuroimaging studies have reported medial OFG/reward and lateral OFG/non-reward and punishment gradient consistently. Some studies also observed elevated lateral OFG activity in the low resilience state such as depression, as well as reduced interconnectivity of the medial OFG (29–31). The theory proposed that lateral OFG/non-reward system might be more easily triggered, and this triggered negative cognitive states, which in turn had positive feedback top-down effects on the OFG/non-reward system. The reward and non-reward systems were likely to operate reciprocally in facilitating the medial OFG/reward system, and they might operate by inhibiting the overactivity in the lateral OFG non-reward/punishment system (9, 31, 32). So increased resilience might be associated with reciprocal interconnectivity between the lateral OFG-related non-reward system and the medial OFG-related reward system.

In addition, given the modulatory roles of psychological resilience, we could find significant positive (i.e., MMSE, Stroop Test, and Digit Span) or negative (i.e., BAI) correlations between clinical symptoms and that resilience is related to the OFG connectivity strength in patient with MCI. Orbitofrontal network might be involved in subjective well-being and active stress coping mediated by psychological resilience (7). Chronic stress was known to have an effect on the transition from MCI to dementia, (33, 34). So maintaining the efficient organization of OFG FC supported neuroflexibility under stress, which might be the intervention strategy for preventing dementia. In actuality, our findings suggested that OFG/HP connectivity and interconnectivities between OFG subregions might be associated with executive/attentive function and anxiety symptoms.

However, contrary to expectations, MADRS scores were positively correlated with FC strength between the OFGs in the high resilient group. This was because despite the working of the resilience related to brain function, older adults with chronic or residual depressive symptoms might be included in this study. Given the characteristics of older adults from geriatric community mental health center, our subjects had relatively chronic and severe depressive symptoms compared with anxiety symptoms or cognitive impairment. Our findings on MADRS score might rather show that high stress levels were accompanied by dynamic brain functions in circuits representing the stress reaction and adaption. In this respect, individuals who failed to show such neuroflexibility in this OFG network could have a high risk of developing dementia. In fact, chronic depression has been well known as a risk factor for dementia (35).

There were several limitations. This study was conducted with a relatively small sample size and a high percentage of female participants. It has been reported that brain FC density might be different according to gender (36, 37). Furthermore, subjects with affective symptoms were included, so this aspect might need to be taken into account to interpret these results as an MCI study (31). These symptoms might interfere with both psychological resilience and cognitive impairment independently. However, independent analyses of depressive and anxiety symptom groups showed that increased OFG FC associated with resilience might be irrespective of brain connectivity related to affective symptoms.

## Conclusion

We demonstrate that psychological resilience may be associated with the orbitofrontal network in the elderly with MCI. Interventions during the pre-symptomatic period of neurocognitive disorder could be effective if they promote the resilience of the brain’s intrinsically efficient arrangement of functional network connections. Understanding of the resilience system modulation of stress responding might be an exciting avenue for future research.

## Data Availability

The datasets for this study will not be made publicly available because due to ethical restrictions, data are available upon reasonable request.

## Ethics Statement

The IRB committee of Ajou University Medical Center approved all protocols of the study (IRB-15-137).

## Author Contributions

Conceived and designed the study: SS, HR, HL, CH. Performed the study: SS, HK, JC, CH. Analyzed the data: BP, JS, N-RK, SS. Wrote the paper: SS, BP.

## Funding

This study was supported by a grant of the Korea Mental Health Technology R&D Project, Ministry of Health & Welfare, Republic of Korea (HI15C0995).

## Conflict of Interest Statement

The authors declare that the research was conducted in the absence of any commercial or financial relationships that could be construed as a potential conflict of interest.
